# Development of Visual Systems for Faces and Objects: Further Evidence for Prolonged Development of the Face System

**DOI:** 10.1371/journal.pone.0099942

**Published:** 2014-06-23

**Authors:** Bozana Meinhardt-Injac, Malte Persike, Günter Meinhardt

**Affiliations:** Department of Psychology, Johannes Gutenberg University Mainz, Mainz, Germany; The University of Western Ontario, Canada

## Abstract

**Background:**

The development of face and object processing has attracted much attention; however, studies that directly compare processing of both visual categories across age are rare. In the present study, we compared the developmental trajectories of face and object processing in younger children (8–10 years), older children (11–13 years), adolescents (14–16 years), and adults (20–37).

**Methodology/Principal Findings:**

We used a congruency paradigm in which subjects compared the internal features of two stimuli, while the (unattended) external features either agreed or disagreed independent of the identity of the internal features. We found a continuous increase in matching accuracy for faces and watches across childhood and adolescence, with different magnitudes for both visual categories. In watch perception, adult levels were reached at the age of 14–16, but not in face perception. The effect of context and inversion, as measures of holistic and configural processing, were clearly restricted to faces in all age groups. This finding suggests that different mechanisms are involved in face and object perception at any age tested. Moreover, the modulation of context and inversion effects by exposure duration was strongly age-dependent, with the strongest age-related differences found for brief timings below 140 ms.

**Conclusions/Significance:**

The results of the present study suggest prolonged development of face-specific processing up to young adulthood. The improvement in face processing is qualitatively different from the improvement of general perceptual and cognitive ability.

## Introduction

Face perception has attracted much attention over the last five decades or more. This interest is partially due to the importance of faces as social and communicative means, although not exclusively; experimental evidence on face perception is far from univocal. One widely accepted fact about face perception is its holistic nature; researchers claim that faces are processed holistically (i.e., in a way that all facial features are glued to form a whole or Gestalt) [1]. Most importantly, the holistic nature of face processing contrasts the part-based processing of common objects, which makes faces “special” [2], [3].

The holistic nature of face perception has been demonstrated repeatedly using context effects and part-to-whole effect as indicators of holistic processing. In the composite face paradigm, a strong and automatic tendency toward holistic face viewing has been demonstrated in which non-attended face halves affect the perception of attended face halves [4], [5]. The effect of unattended facial context is particularly strong when external features (i.e., hair, face, and head outline) are manipulated, and internal features (i.e., eyes, eyebrows, nose, and mouth in their natural configurations) are compared, yielding the illusion of different face identities although two faces share the same internal features [6]–[11].

Similarly, the part-to-whole effect shows that congruent contextual information supports memory and recognition of face parts, compared to seeing these parts in isolation [1]. Both the context and part-to-whole effect are found with faces, but not with non-facial visual objects, which suggests that facial features are embedded into holistic representations and are not stored in isolation. However, presenting faces upside-down strongly impairs face discrimination and reduces holistic face effects [12], [13]. The effect of inversion has been often attributed to impaired processing of second-order or configural relations in inverted faces (i.e., sensitivity to spatial relations among features; for example, inter eye distances), but not only.

Maurer and co-authors suggested that inversion interferes with first-order information and holistic face processing as well [12]. Although the exact mechanisms are poorly understood, the importance of face orientation has often been related to the life-long viewing experience with upright faces that might be taken as a crucial aspect of “face expertise” [14]. In contrast to the face specificity of holistic effects, inversion effects can be found for other classes of visual stimuli, provided that three conditions exist. First, members of the stimulus class have to share the same configuration. Second, members of the class can be individuated based on second-order relational features. Third, subjects have to be experts in using such features to distinguish between members of the same class [14].

Two main groups of findings exist in the examination of the development of face perception. The first group comprises studies stressing the importance of general cognitive factors (i.e., attention, memory) for performance in laboratory face perception tasks. While face perception per se is seen as “special” in terms of faces, but not objects, being processed holistically, the developmental trajectories of face and object processing mechanisms do not differentiate and are both fully developed at the age of 5–6 [15]–[17].

The second line of experimental evidence defines “face specialty” with respect to the *development* of configural and holistic perception, which is not reached until the age of 10 or even later [18]–[22]. Moreover, the proficiency of different face-processing skills seems to be reached at different ages, with the most protracted development being reported for configural information processing [21]–[23]. Recent studies strongly support this claim with evidence for a prolonged development of brain areas involved in face perception and their interconnections [24]–[27]. In a large study including over 60,000 participants, the improvement of face learning ability has been shown to peek just after the age of 30. This finding is the first experimental evidence for late development of face processing that might continue well into the third decade of life [28].

Experimental studies on the development of face perception have rarely included direct comparisons to non-facial objects, and the rare studies that have included comparisons across visual categories have often yielded ambiguous results. For example, studies comparing sensitivity to configural information in faces and objects have led to controversial conclusions about the underlying developmental trajectories [29]–[31]. Overall, it seems that sensitivity to spacing information continues to develop across school-ages; however, handling spacing information seems to rely on general perceptual rather than face-specific mechanisms [31], [32].

Contrary claims suggest early maturity of configural processing mechanisms up to the age of 4 [29], [30]. The contradictory experimental data may likely be due to adaptations of experimental tasks for younger participants (e.g., use of different exposure durations or simultaneous vs. sequential presentation of target and test items) [30], [31]. With respect to holistic face processing, we found similar difficulties in comparing the outcomes of different studies. Ample evidence exists that 3–4 year old children perceive faces holistically, but not non-face objects. Further, some evidence suggests similar and part-based processing of both faces and objects at preschool age [16], [33], [34].

In the present study, we addressed the question of “face specificity” by comparing developmental trajectories of face and object processing across childhood, adolescence, and young adulthood. Accordingly, four age groups spanning an age range from 8–37 years were included in the study: younger children (8–10 years), older children (11–13 years), adolescents (14–16 years), and adults (20–37 years). By comparing different age groups, our aim was to tap into the improvement of the visual processing ability for faces and watches as common non-face visual objects that share complexity and structure with faces [13]. For all age groups, the context congruency paradigm was employed as the experimental task [8], [9], [13]. Participants were required to judge internal features of two faces/watches as same or different, while ignoring the identity of the external features. In this paradigm, the effects of context congruency (for short referred to as “context effect” – see also Methods Section) and orientation were used as measures of holistic and configural face processing, respectively. Variation of exposure duration provided hints on the strength of holistic and configural viewing at different processing stages [8], [9].

Results from previous studies suggest that younger children (8–10 years) and adults differ in the microgenesis of holistic face perception [8], [9], [35]. While adults build holistic face representations almost immediately, children aged 8–10 need between 400–600 msec to do so. However, in these studies, no control with non-face objects was included, which leaves possible alternative explanations for the observed differences between children and adults. Following up on these findings, the aim of the present study was threefold. First, we aimed to find evidence for the improvement in overall matching accuracy for faces and watches across age, and judging its age course. A similar increase in the accuracy for both visual categories could be expected if the face-processing system matures as early as object-related areas, and the development of general cognitive factors alone affects overt task performance. However, if development of the face-processing system is prolonged, a slower increase in matching accuracy could be expected for faces than for watches.

Second, we intended to test, by comparison with non-face objects, whether context and inversion effects are face-specific measures of holistic and configural processing in the 1st and the 2nd decades of life (for adults see [13]). Alternatively, the possible changes in the context effect across childhood and adolescence might reflect improvement in general cognitive factors (e.g., active ignoring and attentional control), while inversion effects might reflect impairment in processing of configural information that is not face specific [21], [31].

Third, we re-examined the claim that developmental changes occur in the microgenesis of the holistic face perception [9]. If age-related differences exist in the time course of holistic processing, the context effect, as a measure of holistic face processing, is expected to be different compared to the adult group when processing at brief, but not longer, times. Hence, the test of whether holistic face processing is fast and instantaneous, as found for adults [8], [9], [35], adds to the current tests of age-related changes in face perception capabilities across development.

## Method

### 2.1 Experimental outline

Tasks and conditions were defined within the framework of the context congruency paradigm [8], [9], [13]. In a same-different matching task, participants were instructed to compare two sequentially presented stimuli with respect to identity or non-identity of the internal features. Internal features included eyes, eyebrows, nose, and mouth in faces and clock-faces in watches. At the same time, participants were instructed to ignore the external features (i.e., hair, face, and head outline in faces and clock-cases in watches). However, if faces/objects are processed holistically, we expected that external features would affect the perception of internal features. Accordingly, external features were manipulated to create congruent and incongruent contexts. In congruent contexts (CC) stimulus pairs were defined in such way that, when the internal features (i.e., attended features) agreed, the external features (i.e., non-attended features) also agreed (same-trial). Likewise, when the internal features disagreed, the external features also disagreed (different-trial).

**Figure 1:**
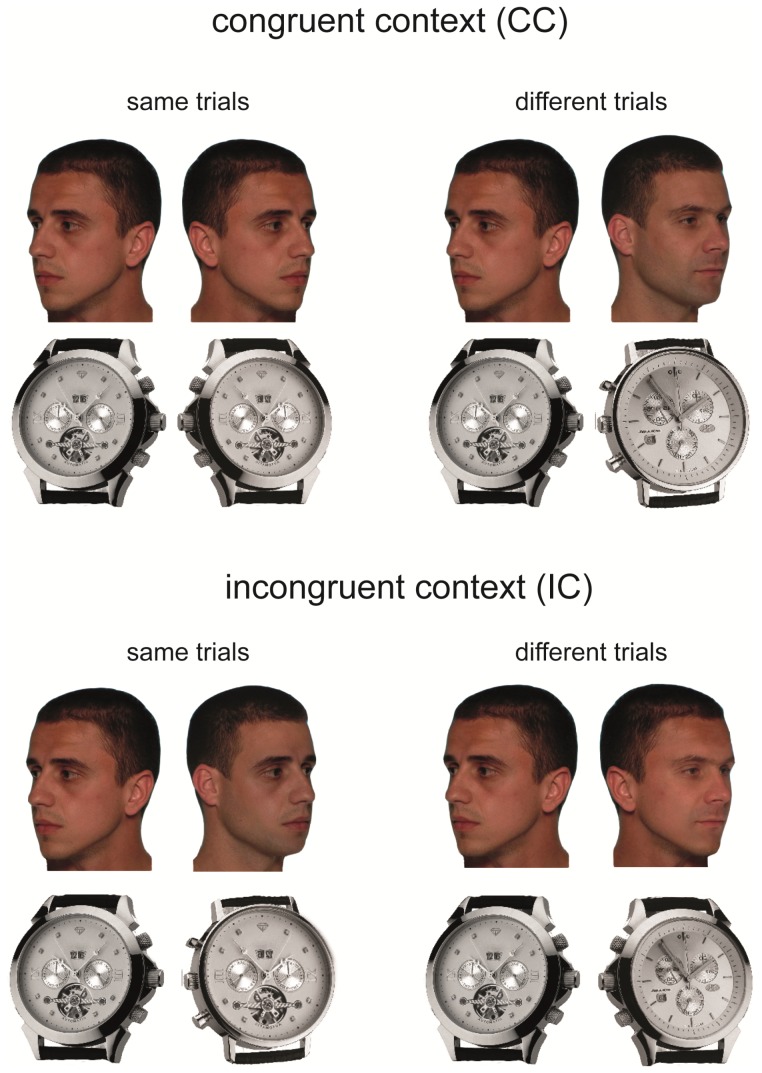
Congruent and incongruent target/no-target feature relationships. In the congruent context condition (CC), the two faces/watches paired in a trial could either be identical or completely different. In incongruent contexts (IC), faces were same in internal features but different in external features and vice versa. Subjects had to judge the identity using only the internal features (inner face/object parts).

In incongruent contexts (IC), the external features disagreed when the internal features agreed (same-trial), and agreed when the target features disagreed (different-trial). Figure 1 illustrates the construction of the stimulus pairs used in same and different trials for CC and IC target feature/context feature relationships. Hence, differences in processing CC and IC trials were measures of holistic processing referred as context congruency effect (context effect).

All stimuli were presented in upright and inverted orientations at six different exposure durations (50, 133, 217, 333, 433, and 633 msec). This large range of timings was included to study early and later face processing stages. Proper duration parameters were found in pilot measurements and have been used in some of our previous studies [8], [9], [13]. Other research groups have used similar timings in studies on the microgenesis of holistic face perception [35].

### 2.2 Stimuli

#### Faces

Professional 3/4 view color studio photographs of male face models were used as templates to create composite face stimuli. We employed 3/4 view photographs in left and right side perspectives; the latter was obtained by mirroring the original left face side photographs. Employing both perspectives of the 3/4 view had the advantage that pixel matching strategies could not be employed, and the observer was left to rely on a true comparison of facial features. Side views had further advantages, such as being better identified and better generalized to other views than the frontal or profile views (for a review see [36]).

The face models had high general resemblance, same hairstyle, and no distinctive single facial features (see [8] for more details about stimuli). Face composites were formed by creating internal feature templates with congruent tracing lines for all stimulus instances using Adobe Photoshop. This procedure maintained near perfect results for the face composites (i.e., original and composite faces were not recognizable as such). Because face stimuli were taken in a photo studio under controlled ambient lighting conditions, the color and hue matching procedures of Adobe Photoshop could be applied successfully to the images. Therefore, the color versions of the images were used because they appeared more natural.

#### Watches

Watch stimuli were selected from internet sources. Because the clock faces and watch cases of all watches were made of steel, the greyscale pictures of watches appeared more natural and were used as stimuli. The images were matched on luminance and contrast. As with the face stimuli, watches were selected in such way that they exhibited general resemblance, showed the same time, and had no particularly distinctive single features. All watch pictures showed watches in a 2/3 view. For the two consecutive presentations in the experimental trials, 3/4 views (faces) and 2/3 views (watches) were shown from the left and right side perspectives. The right side perspective was obtained by mirroring the images at the vertical axis. Because the object features are swapped from left to right in the two mirrored images, pixel region matching strategies are precluded.

### 2.3 Participants

#### Adults

A total of 44 students of psychology from the Johannes Gutenberg University of Mainz participated in the present study (age mean = 23.7; range 20–37). The face-matching experiment included 24 participants participated (19 female), and the watch-matching experiment included 20 participants (all female). The participants of each age group were randomly assigned to either the face-matching experiment or the watch-matching experiment and were given course credit or were paid for their participation.

#### Children and adolescents

A total of 132 subjects participated in the study; 44 from each group: younger children (8–10 years), older children (11–13 years), and adolescents (14–16 years). Table 1 lists the mean ages of each group and number of participants in each experiment.

**Table 1:** Age means of participants in each experiment for two children groups, adolescents, and adults.

Age Group	Face-Matching Experiment		Watch-Matching Experiment	
	N	mean age	N	mean age
8–10	24/19 female	9.2	20/11 female	9.1
11–13	24/11 female	12.3	20/6 female	12.4
14–16	24/12 female	15.1	20/14 female	15.4
adults	24/19 female	23.7	20/all female	23.7

### 2.4 Ethics statement

Prior to the study, all potential participants and parents of participants were informed in writing of the study aims, methods, sources of funding, any possible conflicts of interest, and institutional affiliations of the researcher. Only participants who sent back their written agreement to be contacted about the study were included in the sample. All subjects participated voluntarily. According to the Declaration of Helsinki, written informed consent was obtained from all participants, in case of children and adolescents, consent was also obtained from the parents. The experimental procedures were approved by the local ethics committee at the Johannes Gutenberg University Mainz. Data were analyzed anonymously. The individuals in this manuscript (see Fig. 1) gave written informed consent (as outlined in PLOS consent form) to publish the case details.

### 2.5 Apparatus

The experiment was executed with Inquisit 3.0 runtime units. Patterns were displayed on NEC Spectra View 2090 TFT displays in 1280×1024 resolution at a refresh rate of 60 Hz. The mean luminance of the screen was 75 cd/m^2^. No gamma-correction was used. The room was darkened so the ambient illumination matched the illumination on the screen. Patterns were seen binocularly at a distance of 70 cm. Stimulus patterns and masks subtended 300×400 pixels (width×height). The viewing distance was approximately 70 cm. Participants gave responses on an external keypad.

### 2.6 Procedure

**Figure 2:**
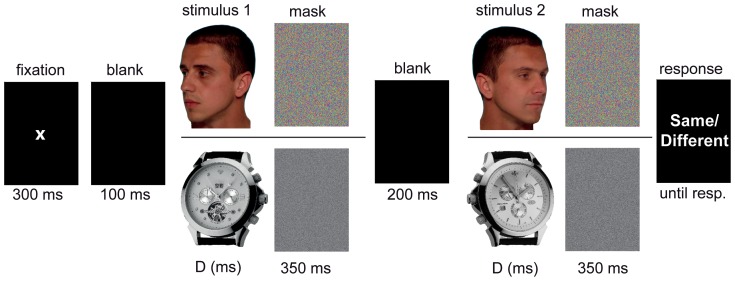
Event sequence of an experimental trial. The upper row illustrates the temporal sequence for faces, the lower for watches.

A same/different matching task was used. Participants were instructed to attend only to the internal features of two consecutively presented stimuli and judge their identities. The temporal order of events in a trial sequence was as follows: fixation mark (300 msec) - blank (100 msec) - 1st stimulus frame (Duration) - mask (350 msec) - blank (200 msec) - 2nd stimulus frame (Duration) - mask (350 msec) - blank frame until response (see Fig. 2). Masking of the stimulus frames was done with scrambled stimulus patterns at a tile size of 10 pixels. The presentation positions of each of the two face images were shifted by 20 pixels away from the center in a random direction to preclude focusing on the same image parts in consecutive presentations.

For watches, one of the two stimulus instances of a trial was scaled down to 70% of its original size. The main reason for using size variation for watches was that, by doing this, the performance for watches and faces matched the longest exposure duration for upright presentation and in congruent contexts (baseline to equate overall performance level of faces and watches. The scaling factor necessary to equate baseline performance for faces and watches was calibrated in pilot measurements before the main experiment started.

Acoustical trial-by-trial feedback about correctness was given by brief headphone tone signals (correct: “tack”-tone, incorrect: “tacktack”-tone). Each participant completed 2 (orientation: upright/inverted)×2 (context: congruent/incongruent)×2 (trial type: same/different)×6 (exposure duration: 50, 133, 217, 333, 433, 633 msec)×16 (replication) = 768 trials. These trials were presented at random intervals, but in 3 blocks of 256 trials. One block took about 12 minutes.

All participants were made familiar with the task prior to the main experimental blocks. Adults went through 8 minutes of randomly selected probe trials to ensure they understood the instructions and could put them into practice. Children and adolescents were carefully prepared for the experiment. First, they were explained the matching task with alternating views using printed examples of the stimuli. Afterwards, the children went through the same experimental practice trails as did the adults. After the preparation phase, the experimental blocks were administered to each participant with interleaved pauses [9].

### 2.7 Performance measures

Accuracy of responses was measured by calculating the proportion of correct rates from both correct same and correct different responses. This process allowed the accuracy measure to be devoid of a possible bias toward one or both response categories, and was a concrete and highly intuitive measure of performance. Each proportion correct datum rested on *N* = 32 trials. Sixteen of these were same-trials, and 16 were different-trials.

To assess possible response bias toward either “same” or “different” responses, we calculated a concrete measure that directly reflected which of both response categories was preferred. In a same/different experiment the “same” response category is usually defined as the target category. Accordingly, the hit rate [Hit, P(“same”|same)] and miss rate [Miss, P(“different”|same)] referred to the rates of being correct/incorrect on same-trials. The correct rejection rate [Hit, P(“different”|different)] and false alarm rate [FA, P(“same”|different)] were the corresponding rates for being correct/incorrect on different-trials. The proportion measure
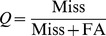
indicates which of the two kinds of errors is more likely. If Q = 0.5, then both kinds of errors are made with the same frequency; Q>0.5 indicates more misses and Q<0.5 indicates more false alarms. The bias measure Q has the advantage that it is more concrete than the response criterion *c* derived from signal detection theory, which is also used in the literature. For example, a value of Q = 0.7 means that 70% of all errors are wrong “different” responses and 30% are wrong “same” responses.

## Results

### 3.1 Proportion correct as a function of exposure duration

**Figure 3:**
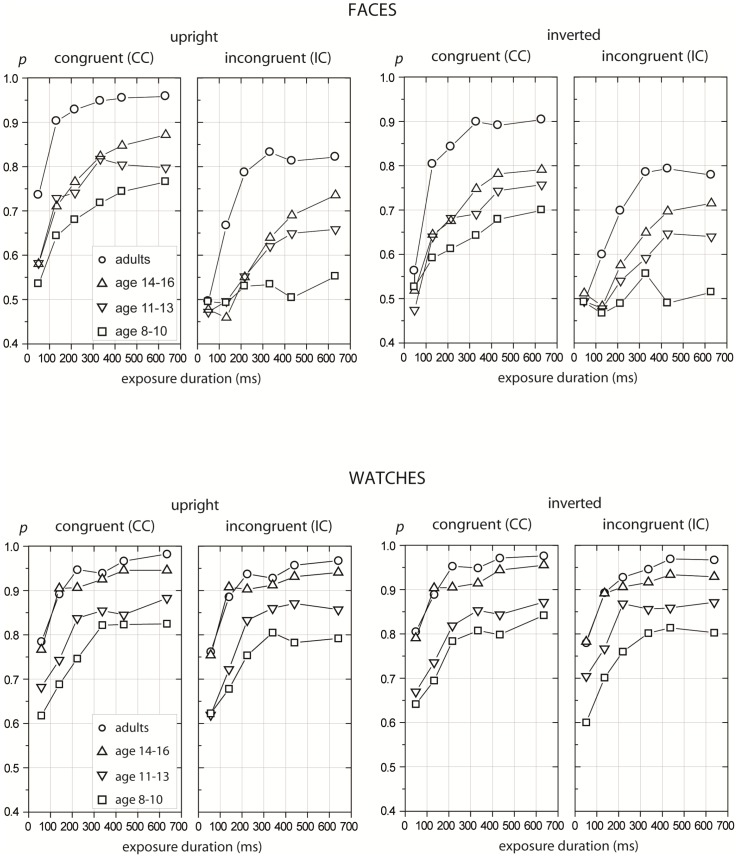
Proportion correct as a function of exposure duration. Mean proportion correct rates for adults and children (ages: 8–10, 11–13, and 14–16) as a function of exposure duration for matching faces (upper panels) and watches (lower panels) in upright (left) and inverted (right) orientations and at the two levels of context congruency (congruent vs. incongruent).

Figure 3 shows the proportion correct measure as a function of exposure duration for each experimental condition and for all four age groups tested (adults: 20–37 years; adolescents: 14–16 years; older children: 11–13 years; younger children: 8–10 years). Data points indicate participants' mean values.

As evident at first glance, major differences existed between adult participants and children/adolescents in the overall level of matching performance with both face and watch stimuli. Overall, adults reached higher accuracy rates compared to all three age-groups of children/adolescents in all experimental conditions. However, this advantage seemed to be stronger with faces than with watches. At the same time, matching performance for faces, but not watches, was strongly modulated by the effects of context and inversion.

To assess the effects of all sources of variation, the proportion correct data were analyzed using repeated measures ANOVA with stimulus type [2; faces vs. watches] and age [4; adults (20–37 years); adolescents (14–16 years): older children (11–13 years) and younger children (8–10 years)] as between-participant factors and context [2; congruent vs. incongruent], orientation [2; upright vs. inverted], and exposure duration [50, 133, 217, 333, 433, and 633 msec] as within-participant factors. All ANOVA results are listed in Table 2.

**Table 2:** Repeated measurements ANOVA.

	SS	*df*	MS	F	*p*
**{1} stimulus**	**33.15**	**1**	**33.15**	**216.29**	**<0.001**
**{2} age**	**20.72**	**3**	**6.91**	**45.06**	**<0.001**
**stimulus×age**	**1.24**	**3**	**0.41**	**2.69**	**0.048**
**{3} context**	**5.69**	**1**	**5.69**	**254.16**	**<0.001**
**context×stimulus**	**4.51**	**1**	**4.51**	**201.16**	**<0.001**
context×age	0.03	3	0.01	0.47	0.704
context×stimulus×age	0.01	3	0.00	0.13	0.943
**{4} orientation**	**0.37**	**1**	**0.37**	**50.69**	**<0.001**
**orientation×stimulus**	**0.64**	**1**	**0.64**	**88.66**	**<0.001**
orientation×age	0.05	3	0.02	2.52	0.060
**orientation×stimulus×age**	**0.06**	**3**	**0.02**	**2.69**	**0.048**
**{5} time**	**21.09**	**5**	**4.22**	**297.80**	**<0.001**
**time×stimulus**	**0.35**	**5**	**0.07**	**5.00**	**<0.001**
**time×age**	**0.76**	**15**	**0.05**	**3.57**	**<0.001**
**time×stimulus×age**	**1.45**	**15**	**0.10**	**6.81**	**<0.001**
**context×orientation**	**0.25**	**1**	**0.25**	**44.39**	**<0.001**
**context×orientation×stimulus**	**0.25**	**1**	**0.25**	**43.58**	**<0.001**
**context×orientation×age**	**0.06**	**3**	**0.02**	**3.33**	**0.021**
context×orientation×stimulus×age	0.03	3	0.01	1.61	0.188
**context×time**	**0.23**	**5**	**0.05**	**7.33**	**<0.001**
**context×time×stimulus**	**0.44**	**5**	**0.09**	**13.80**	**<0.001**
**context×time×age**	**0.35**	**15**	**0.02**	**3.64**	**<0.001**
**context×time×stimulus×age**	**0.22**	**15**	**0.02**	**2.31**	**0.003**
orientation×time	0.02	5	0.01	0.93	0.461
orientation×time×stimulus	0.01	5	0.00	0.56	0.730
orientation×time×age	0.09	15	0.01	1.19	0.276
orientation×time×stimulus×age	0.09	15	0.01	1.13	0.328
context×orientation×time	0.04	5	0.01	1.43	0.211
context×orientation×time×stimulus	0.02	5	0.01	0.92	0.470
**context×orientation×time×age**	**0.14**	**15**	**0.01**	**1.84**	**0.026**
1×2×3×4×5	0.09	15	0.01	1.11	0.341

Note: The results of repeated measurements ANOVA with context (congruent vs. incongruent), orientation (upright vs. inverted) and exposure duration (“time”: 50, 133, 217, 333, 433, and 633 msec) as within-subject factors, and age (adults, 8–10, 11–13, and 14–16 years) and stimulus type (“stimulus”: faces vs. watches) as between-subject factors. Significant main effects and interactions are marked boldface.

The five main effects (i.e., stimulus type, age, context, orientation, and exposure duration) were all highly statistically significant. Matching performance improved with increasing age and prolonged exposure duration and were overall higher for watches than for faces, for congruent vs. incongruent contexts, and in upright vs. inverted orientation. Furthermore, the findings yielded strong interactions between stimulus type and measures of holistic and configural processing (stimulus type×context; stimulus type×orientation; stimulus type×orientation×context congruency).

A Fisher LSD post hoc test revealed no significant effects between upright and inverted or between congruent and incongruent watch stimuli (all *p*>.11); both effects were strongly significant for faces (all *p*<.001). This finding indicates different processing regimes of face and watch stimuli [13]. However, the present study focuses on age-related differences in overall performance depending on stimulus class (**A**) as well as potential age-related differences in holistic measures (**B**).

#### (A) Do age-related differences exist in performance with respect to stimulus class?

**Figure 4:**
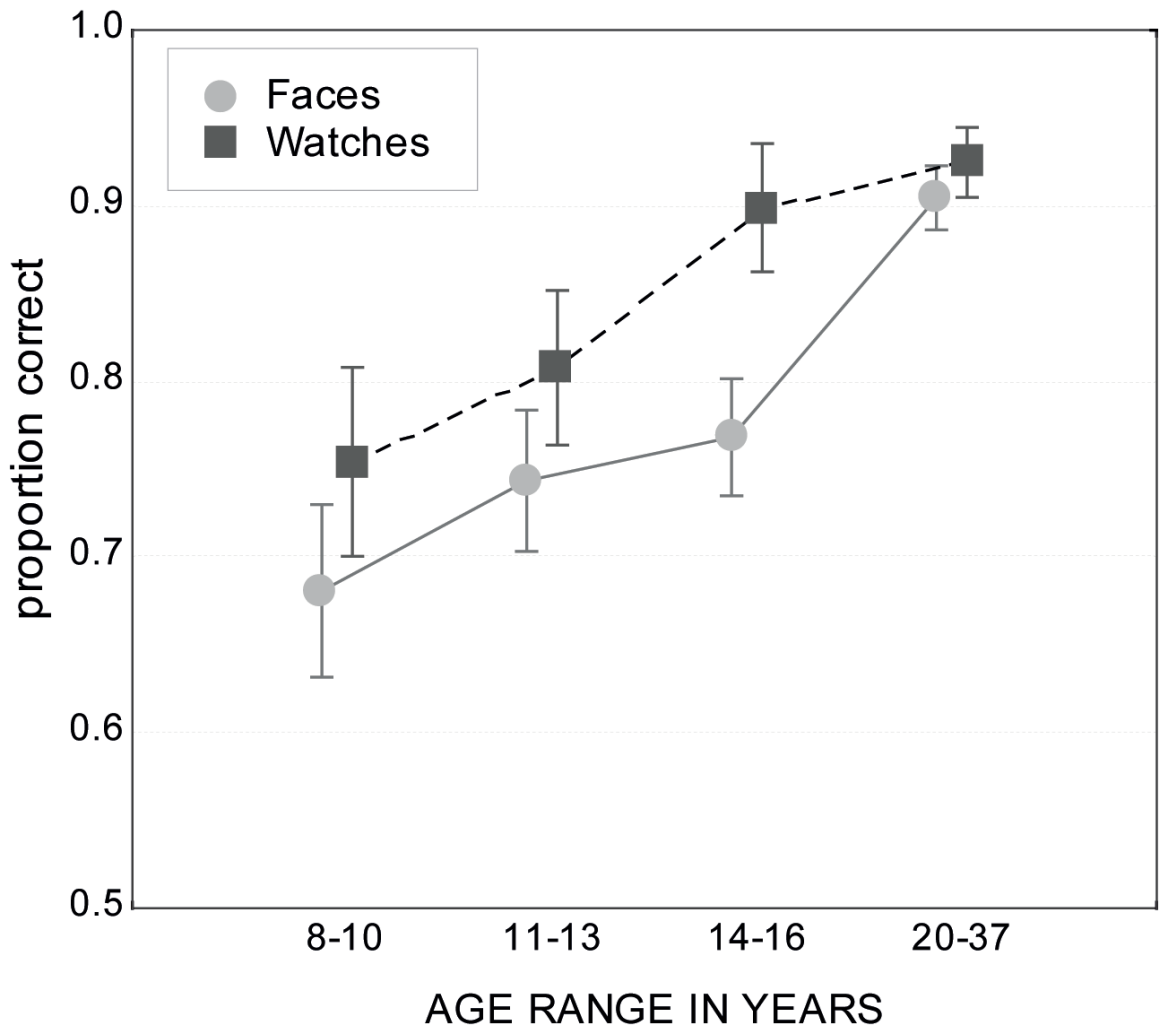
Overall object matching performance. Mean proportion correct rates for performance with upright stimuli and congruent contexts (totally the same or totally different objects) for faces and watches, agglomerated over exposure duration.

This question can be answered by comparing performance in the baseline condition (upright objects and congruent trials) between both stimulus types at any age, and by comparing baseline-performance of children/adolescents against the performance of young adults. For this purpose, a two-way ANOVA with the factors age and stimulus type was conducted with the mean proportion correct data (across exposure durations) for congruent trials with upright presentation. The data are shown in Fig. 4.

Both main effects and their interaction were statistically significant [main effect of stimulus type: *F*(1,42) = 29.46, *p*<.001; main effect of age: *F*(3,126) = 37.76, *p*<.001; stimulus×age: *F*(3,126) = 2.75, *p*<.05)]. A Fischer LSD post hoc test revealed that, for both children groups and adolescents, baseline performance was significantly better with watches than with faces (all *p*<.01); however, in adults, no significant differences existed in baseline performance for faces and watches (*p* = .45). Furthermore, a Fischer LSD post hoc test revealed significant differences in baseline performance between adults and all other age groups when faces were used as stimuli (all *p*<.001). With watches, 8–10 and 11–13 year-old children were significantly less accurate than were adults (both *p*<.001), but not adolescents (*p* = .36).

In sum, the data show that in both children groups and adolescents, the performance with watches was significantly more accurate than with faces. With faces, adolescents did not reach the performance of young adults, but did reach it with watches (see also Fig. 4). Hence, adult levels of performance were reached earlier with watches, which indicates a face-specific developmental delay.

#### (B) Do age-related differences exist in holistic face perception?

For this question, interactions involving age, context, and orientation were relevant. First, the two-way interactions, age×context and age×orientation, were not statistically significant (*p* = .70 and *p* = .06, respectively, see Table 2), which indicates the same magnitude of holistic and configural face processing at all ages. However, the age×orientation interaction failed to reach significance, and there were a few more significant interactions, which suggests that age-related effects exist not only in overall performance (interaction age×stimulus), but also in measures of holistic and configural processing: three-way interactions age×context×orientation and age×stimulus×orientation. The data pattern was made more complicated by further significant interactions between age×context×exposure duration and age×stimulus×context×exposure duration (see Table 2 and Fig. 3).

To simplify the relations among age and measures of holistic and configural processing with their dependence on temporal resources, we calculated the context and inversion effects at short (50/133 ms) and long (433/633 ms) exposure durations. Context effects were calculated as the accuracy difference between congruent and incongruent trials presented in upright orientation, whereas inversion effects were calculated as the accuracy difference between upright and inverted stimuli in the congruent condition for both stimulus types (see Fig. 1). By focusing on short and long exposure durations for the two independent image manipulations, we directly tested whether differences existed in the early and later processing stages of faces and objects between different age groups.

### 3.2 Context effects

**Figure 5:**
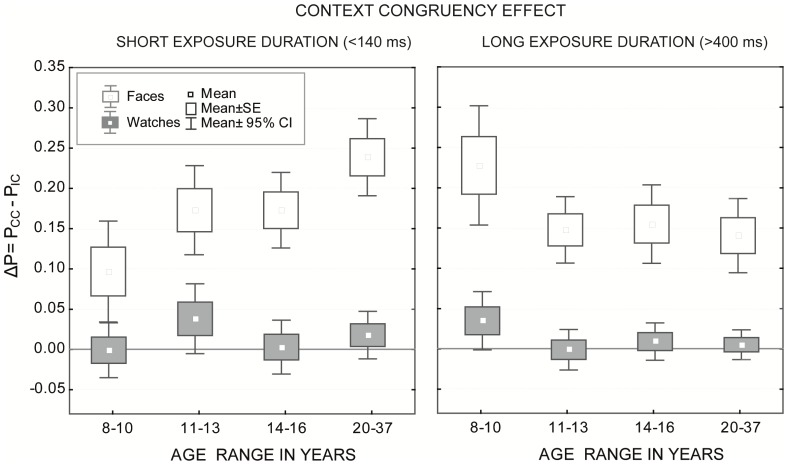
Context effects. Mean accuracy differences for congruent (CC) and incongruent (IC) contexts of upright presented faces and watches at short (<140 ms) and long (>400 ms) exposure durations. The Box-Whiskers indicate the mean with standard error (box) and confidence intervals (whiskers). An effect was significant if 0 was outside the confidence interval.

The context effect was calculated as the accuracy difference obtained for congruent and incongruent contexts (Δ*P* = *P_CC_−P_IC_*) at the individual level. The data obtained for upright presented faces and watches at the two shortest (50 and 133 msec) and two longest exposure durations (433 and 633 msec) were merged by averaging the individual level for upright trials only. These data were analyzed using an ANOVA with exposure duration (2; short vs. long) as the within-subjects factor and stimulus type (2; faces vs. watches) and age (4; young adults, adolescents, younger, and older children) as the between-subjects factors. An overview of the results is given in Fig. 5.

The ANOVA revealed a significant main effect for stimulus type (*F*(1, 168) = 162.16, *p*<.001), which indicates larger context effects for faces than for watches. The main effects of age and exposure duration were not statistically significant. However, significant interactions existed for age×exposure duration (*F*(3, 168) = 9.16, *p*<.001) and age×exposure duration×stimulus type (*F*(3, 168) = 3.54, *p*<.05). For watches, a Fisher LSD post-hoc test revealed no significant differences in the strength of context effects at any age group or exposure duration (all *p*>.18). In fact, judging whether 0 was within the confidence interval of the mean differences showed no significant context effects at all ages and all exposure durations (see gray Box-Whiskers in Fig. 5). This finding suggests that participants were able to focus their attention on only the internal parts of the watch-stimuli, and they performed equally well in matching the incongruent and congruent stimulus pairs.

With face stimuli, pronounced differences existed in the strength of the context effect related to age and exposure duration (see Fig. 5). At the short exposure duration, significant differences existed in the strength of the context effect between 8- to 10-year-old children and all other age groups (all *p*<.01). Additionally, no significant differences were found between older children (11–13 years) and adolescents (14–16 years); however, a significant difference existed for both children and adolescents compared to adults (both *p*<.05). At the long exposure duration, 8 to 10 year olds differed from all other age groups (all *p*<.01); however, no significant differences were found between the groups of older children (11–13 years) and adolescents (14–16 years) to each other or to the adult group (all *p*>.63).

### 3.3 Inversion effects

**Figure 6:**
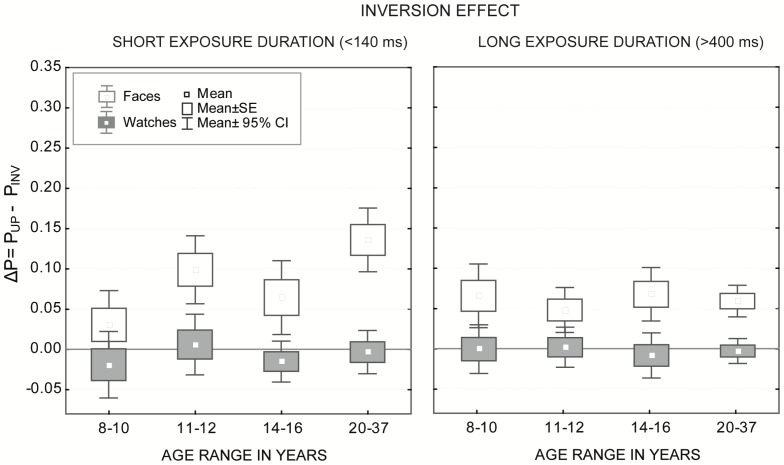
Inversion effects. Mean differences of performance for upright and inverted stimuli for faces and watches at short (<140 ms) and long (>400 ms) exposure durations. The Box-Whiskers indicate mean with standard error (box) and confidence intervals (whiskers). An effect was significant if 0 was outside the confidence interval (CI).

Inversion effects were calculated as difference measures (Δ*P* = *P_UPR_−P_INV_*). Again, the data were merged by averaging the measurements at the two shortest and two longest exposure durations for CC at the individual level. The data were analyzed by ANOVA. A results overview is given in Fig. 6.

Overall, the analysis revealed a significant main effect for stimulus type (*F*(1, 168) = 82.88, *p*<.001) and a significant interaction for age×exposure duration (*F*(3, 168) = 3.28, *p*<.05). No other effect reached statistical significance. For watches, a Fisher LSD post-hoc tests revealed no significant inversion effect at any age group or exposure duration (all *p*>.31). As found for the context effects, no significant inversion effects existed at any age tested or for brief and long timings (see Fig. 6: 0 was within all confidence intervals indicated by the gray Box-Whiskers).

The data pattern for faces was more complex and clearly different for short and long exposure durations. At the short exposure duration, a significant difference was found between different age groups, whereby the inversion effect in younger children (8–10 years) was smaller than that in older children (11–12 years) and adults (both *p*<.001), and failed to reach significance when contrasted to adolescents (*p* = .09). Further, no significant difference existed in the inversion effect between older children and adolescents (*p* = .12); however, both groups differed significantly from adults (both *p*<.05). At the long exposure duration, no significant age differences were found, and the inversion effect was of comparable magnitude between all age groups (all *p*>.10).

### 3.4 Response bias

**Figure 7:**
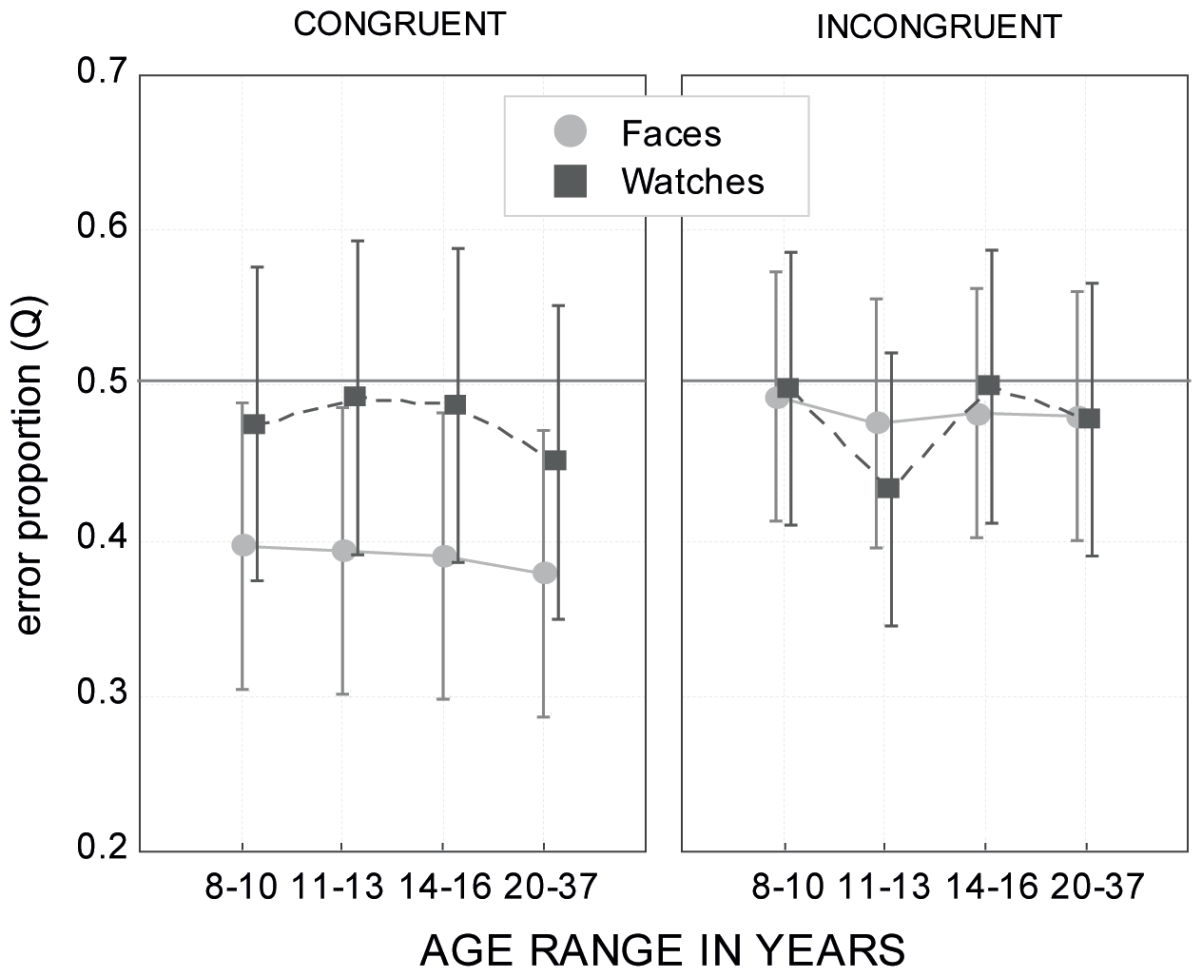
Response bias. The bias measure Q, defined as the proportion of incorrect “different” responses to incorrect “different” and incorrect “same” responses. Values of Q>0.5 indicate a bias toward “different” responses, and values of Q<0.5 indicate a bias toward “same” responses. Error bars indicate 95% confidence intervals of the means.

The bias measure Q data (see Section *Performance measures*) was analyzed using the same ANOVA procedure as the original proportion correct data except only short (50 and 133 msec) and long (433 and 633 msec) exposure durations were included (see above). Hence, the repeated measures ANOVA was calculated with age (4; age groups) and stimulus type (2; faces vs. watches) as between-subject factors and context (2; congruent vs. incongruent), orientation (2; upright vs. inverted), and exposure duration (2; short vs. long) as within-subject factors. The data are shown in Fig. 7.

The main effects of stimulus [*F*(1, 168) = 8.64, *p*<.01] and context [*F*(1, 168) = 29.42, *p*<.001] and their interaction [*F*(1, 168) = 28.37, *p*<.001] were statistically significant. Importantly, the main effect of age was not statistically significant [*F*(3, 168) = .54, *p* = .65], nor were there significant two-way or higher interactions involving age. The main effects of orientation and exposure duration did not reach statistical significance [*F*(1, 168) = 3.07, *p* = .08 and *F*(1, 168) = 1.5, *p* = .22, respectively].

In sum, the response bias analysis revealed no differences in response strategies between different age groups. Overall, a tendency existed toward “same” responses for face stimuli in congruent context (i.e., totally same/different trials), but no response bias was obtained for incongruent trials (see Fig. 7). For watches, there was practically no response bias across the experimental conditions.

## Discussion

In the present study face and non-face object processing was compared in younger children (8–10 years), older children (11–13 years), adolescents (14–16 years), and adults (20–37 years). The results revealed a continuous increase in matching accuracy for faces and objects (i.e., watches) across childhood and adolescence. However, this increase was different for both object categories. Adult levels in face matching performance were not reached in the adolescents group, while with watches, adults and adolescents performed equally well. Effects of context and inversion, used as measures of holistic and configural processing, were clearly restricted to faces at any age tested. This finding corroborates that both measures tap into face-specific processing mechanisms that are not shared with other objects. The results also indicate that face and object perception relies on different processing regimes from the age of 8 to adulthood, probably starting from an earlier age [33]. Accordingly, the prolonged development of face processing abilities reported here cannot be reduced to more general cognitive or perceptual factors as suggested elsewhere [15].

With increasing age, there was great improvement in using holistic and configural cues in face perception. The age differences in context and inversion effects were particularly strong at brief timings, where mostly early, preattentive processing stages are involved. Because response strategies, as captured by response bias, did not differ for different age groups or for short and long exposure durations, the age effects are rather perceptual than decisional in nature. Altogether, these results provide evidence for prolonged development of face-specific processing up to adolescence that is not shared by other visual objects. Mechanisms involved in face processing are clearly different from those involved in object processing, and a specific developmental trajectory exists for each visual category. In the following, we discuss these main findings more in detail.

The results of the present study demonstrate improvements of object matching accuracy for face and non-face objects across childhood. Adults' accuracy with non-face common objects (watches) is reached in the adolescence group at the ages of 14–16. However, with faces, even adolescents performed less accurately than did adults, which suggests that the mechanisms involved in face processing develop well into the second decade of life. The age-differences in the overall performance found in the present study fit well with the existing data on face [9], [20], [22], [23], [27], [37] and object perception [31], [32], [38]. Thus, we add to the notion that adult-like performance in visual tasks is not reached until adolescence.

Nonetheless, the question of how much of this prolonged development is either due to qualitative changes in visual processing or to the improvement of general cognitive factors (e.g., memory or attention) with age is still a matter of discussion. This is especially true in the field of face perception where the issue of developmental trajectories of face specific versus general perceptual mechanisms is controversial [15]. With respect to this ongoing debate [9], [15], [16], [20], [23], [36], the present study provides new evidence supporting the claim that improvement in face perception is qualitative in nature and clearly distinct from improvement in object perception.

Despite the large body of literature on the topic, researchers have made few attempts to study the development of face and non-facial objects in parallel [29], [33]. Mostly, studies on the development of face perception have focused only on faces and have typically ignored findings on the development of object processing. Although non-face object processing is assumed to be mature early, the development of the underlying mechanisms might be prolonged into the teenage years, at least when an analysis of metric or configural relations among object parts is considered, which are relevant for complex objects [31], [32]. On the other hand, recognition of feature object properties seems to be as accurate as adults at the ages of 7–8 [32], [38]. This finding indicates different developmental trajectories for configural and part-based comparisons.

Whereas everyday objects are processed at the categorical level (i.e., as a table or a cup), faces are processed at the individual level (i.e., faces are identified as belonging to a certain person). Because all faces share the same features with respect to first-order relations, identification requires reliance on second order, metric-configural facial properties [21], [39]. Similar mechanisms in object processing are automatically used only by experts (e.g., dog experts [14]). Hence, the strength of metric-configural processing (often measured by inversion effects) can provide hints for the use of these cues in both objects and faces [14].

In the present study, inversion effects were clearly restricted to faces at all ages tested. The lack of use of configural cues was not surprising because participants were no experts in watches. Note that expertise is not gained through daily use of watches for telling the time, rather only by learning to individuate many different watch exemplars [14], [39]. Additionally, the current results show the strongest reliance on configural information in adults at the short exposure duration (<140 msec). Even in adolescents, inversion effects were not as strong as in adults (see Fig. 6 and Section 3.3.). However, the age-related differences vanished at the long exposure duration (>400 msec).

The inversion effect were calculated as the difference between upright and inverted congruent faces, which means that young children performed at the level of about 70% and adults were at the range of about 90% correct responses at the long exposure durations (see Fig. 3). Hence, it cannot be excluded that the ceiling performance of adults with both upright and inverted faces at the long exposure duration precluded some age differences in the strength of the inversion effect. However, these differences (even between adults and adolescents) at the short exposure duration concern automatic and fast processing of configural relations in adult face perception [18], [19], [21], [23]. Because the effect was obtained only with faces, not with watches, it can be taken as an indicator of the high expertise in adult face perception that is still developing in adolescence [37]. The data on the microgenesis of the holistic facial percept supports this view.

Although children and adolescents exhibit holistic face processing, the findings revealed pronounced differences in the strength of context effects with respect to viewing time. For early feed-forward processing at brief timings, precluding saccades and serial scan (<133 msec), the context effect was smaller in both children groups and the adolescents group compared to the adult group. At long exposure durations, the context effect was very pronounced for the younger children group (8–10 years, whereas no differences emerged between older children (11–13 years), adolescents (14–16 years), and adults. Thus, the data showed significant age differences in the measure of holistic viewing at early, but not at later, processing stages. While adults perceive faces holistically virtually from stimulus onset [9], [35], children require some additional time to build the same holistic representations.

The microgenesis of holistic face perception speeds up across childhood and adolescence, but does not reach adult levels even at the ages of 14–16. No similar effects were obtained for watches at any age tested, which is in line with previous studies in which holistic effects (e.g., the composite and context effects) were not found for non-facial objects [33]. It seems plausible that the increase of holistic vision reflects at least some aspects of specialization based on daily experience in face individuation [39]. Internal facial features are particularly important to individuate faces in adults [40], [41]; however, play only a marginal role in child and adolescent face recognition [9], [42], [43]. Thus, the increase in efficiency of holistic face mechanisms most likely underlies overall improvement in tasks that require face perception and identification and stronger reliance on internal facial features with increasing age.

Based on the data of the present study, we conclude that the improvement in face perception is prolonged well into second decade of life. It is clearly distinctive from the development of general cognitive or perceptual abilities. This change concerns improved encoding of configural and holistic face properties particularly in early, automatic face processing, and is related to the switch toward using internal facial features for identification [44], [45].

Neuroimaging data strongly support the notion that face processing changes qualitatively with age as such data have provided evidence that cortical areas specialized for face perception develop gradually during the first two decades of life [46]–[48]. Changes in functional connectivity patterns within face processing networks may account for the differences in the microgenesis of holistic and configural face perception involving early processing stages, as demonstrated here and elsewhere [24]–[27].
